# Assessing the association between quantity and quality of family caregiver participation in decision-making clinical encounters on patient activation in the metastatic breast cancer setting

**DOI:** 10.1007/s00520-024-08593-1

**Published:** 2024-06-11

**Authors:** Nicole L. Henderson, Tanvi Padalkar, Garrett Bourne, Emma K. Hendrix, Courtney P. Williams, J. Nicholas Odom, Kristen Triebel, Gabrielle B. Rocque

**Affiliations:** 1 School of Medicine, University of Alabama at Birmingham, Birmingham, AL USA; 2 School of Nursing, University of Alabama at Birmingham, Birmingham, AL USA

**Keywords:** Cancer, Oncology, Family caregivers, Decision-making, Patient activation, Treatment planning, Satisfaction with care, Caregiving

## Abstract

**Objective:**

Caregivers support individuals undergoing cancer treatment by assisting with activities, managing care, navigating healthcare systems, and communicating with care teams. We explored the quality and quantity of caregiver participation during recorded decision-making clinical appointments in women with metastatic breast cancer.

**Methods:**

This was a convergent parallel mixed methods study that utilized qualitative and quantitative data collection and analysis. Caregiver participation quality was operationalized using a summative thematic content analysis to identify and sum caregiver actions performed during appointments. Performance of a greater number of actions was considered greater quality of participation. Caregiver participation quantity was measured by calculating the proportion of speaking time. Participation quality and quantity were compared to patient activation, assessed using the Patient Activation Measure 1-month post decision-making appointment.

**Results:**

Fifty-three clinical encounters between patients with MBC, their caregivers, and oncologists were recorded. Identified caregiver actions included: General Support; Management of Treatment or Medication; Treatment History; Decision-Making; Insurance or Money; Pharmacy; Scheduling; Travel Concerns; General Cancer Understanding; Patient Specific Cancer Understanding; Caregiver-Initiated or Emphasis on Symptom Severity; and Caregiver Back-Up of Patient Symptom Description. Caregivers averaged 5 actions (SD 3): 48% of patient’s caregivers had low quality (< 5 actions) and 52% had high quality (> 6 actions) participation. Regarding quantity, caregivers spoke on average for 4% of the encounter, with 60% of caregivers speaking less than 4% of the encounter (low quantity) and 40% of caregivers speaking more than 4% (high quantity). Greater quality and quantity of caregiver participation was associated with greater patient activation.

**Conclusions:**

Caregivers perform a variety of actions during oncological decision-making visits aiding both patient and provider. Greater participation in terms of quantity and quality by the caregiver was associated with greater patient activism, indicating a need for better integration of the caregiver in clinical decision-making environments.

## Introduction

Caregivers perform pivotal roles in assisting with the care of individuals undergoing treatment for cancer. Caregivers provide a broad range of assistance within caretaking as they assume multiple responsibilities during patient’s cancer treatment [1]. They manage the logistics of financial support, travel, appointments, navigating health care systems, disease monitoring, and treatment administration [2–8]. Alongside this, caregivers are actively involved in the discussion on symptoms and the treatment plan during consultation communications [4, 9, 10], in shaping treatment adherence [11, 12], and in facilitating healthy behaviors [12]. Underlying this, caregivers are crucial in providing emotional and psychosocial support [1, 13, 14]. Caregivers take on decision-support roles by assisting with information about cancer, in understanding and processing disease-related details, and in decision-making about the initiation or stopping of treatment [13, 15–17]. These actions may be more extensive and dynamic in advanced illness conditions such as metastatic breast cancer (MBC), as the longer disease course and repeated instances of treatment changes result in multiple opportunities for care-related decisions [16, 18]. Beyond the type, timing, and location of treatment, decisions also include logistical and financial concerns, attending appointments, and getting access to treatment [19–21].

The impact of cancer caregivers’ on patients’ decision-making can be both positive and negative. In a nationwide sample of over 5200 newly diagnosed cancer patients, nearly half indicated that they shared treatment decision-making responsibility equally with a partner or another family member, and one in five solicited insight from someone close to them [16, 22]. In their systematic review, Cincidda and colleagues highlight that caregivers preferred a collaborative or passive role in decision-making with patients giving significance to caregiver preferences in decision-making [23–26]. Caregivers are referred to as conductors of information from patient to clinician, facilitators, and aids in considering treatments [27]. Caregiver involvement as associated with patient activation has also been associated with increased treatment satisfaction and adherence [19, 21, 28–31].

Researchers have found caregiver involvement to be highly influential on patient self-management behaviors, self-efficacy, stress, and depression [32–34]. Additionally, caregiver health literacy affects treatment outcomes and self-care behaviors in patients with cancer, and they may employ this comprehension of health information and services when making decisions [35–38]. Caregivers are often individuals of high importance and trust to the patient, meaning that they may have unparalleled insight into the patient’s life and can champion problems and preferences expressed by the patient outside of the clinical setting [39]. Increased partner-caregiver involvement has been associated with lower patient decision regret for breast cancer patients and improved subjective decision quality and deliberation [40, 41].

At the same time, greater involvement of the caregiver in the clinical encounter could have negative consequences. Caregivers may overshadow the patient and heighten their distress by pushing for treatment options that are inconsistent with the patient’s personal values [16, 22, 42, 43]. Patients may not feel comfortable to disclose sensitive aspects of their symptoms in the caregiver’s presence [44]. On the other hand, caregivers who are under stress, burdened, or facing physical health issues may lack the emotional or cognitive ability to engage in the decision making processes [45]. Information overload may impact caregiver’s ability to support patient decision making efforts and caregiving outcomes [46]. Further, consideration of the caregiver role as an obligation rather than a choice is associated with communication difficulty with the patient and patient minimization of the caregiver role in decision making [47]. Past research has underscored that caregiver involvement is associated with patients’ prioritizing length of life over quality of life in making treatment decisions [48]. Discordance in caregiver shared decision making has been associated with conflicts in relationships and a lack of awareness and communication regarding each other’s care preferences [49].

Laidsaar–Powell and colleagues highlight this variability in the caregivers’ interaction in clinical encounters as variable and ranging from “active partner” to “welcome guest” to “intruder” [4]. Family caregivers, whose identities are viewed as inextricably relational with the patients, may play a role in co-editing the patient’s future self through treatment decision making [50]. In end-of-life oncological situation, researcher has identified family members involved in decision making were regarded as “second patients” [51]. Greater evaluation of the real-world implications of the crucial interaction between patients and caregivers in clinical settings is needed, despite existing studies on decision-making involvement preferences and survey-based evaluations of patient activation by caregiver involvement [1, 42, 52–56].

Little is known about the range of caregivers’ actions during clinical encounters and how these may affect patient activation—defined as the skills, knowledge, and confidence to manage one’s own health as an active participant [57]. Higher patient activation has been associated with positive care experiences, uptake of self-management behaviors, and improved outcomes [58]. Because caregivers often play extensive roles in the patient’s cancer trajectory, it is crucial to delve into the impact of caregiver interactions on patient engagement during clinical encounters. Therefore, this study sought to characterize caregiver involvement during treatment decision-making visits and its association with patient activation for women with metastatic breast cancer.

## Methods

### Study design and sample

We used a convergent parallel mixed-methods design, which involves the concurrent collection, analysis, and presentation of both qualitative and quantitative data in order to better understand the complexities of caregiver participation in clinical decision-making appointments. In this case, we incorporated the qualitative analysis of recorded clinical decision-making encounters in women with MBC and the quantitative analysis of timecoding and patient surveys to explore the effect of caregiver participation on patient activation.

This constituted a sub-study within a larger randomized control trial (RCT) evaluating the impact of formalized shared treatment decision-making planning on women with metastatic breast cancer (NCT 03806738). The intervention included presenting patient-reported data within the context of decision-making. Patients participating in the parent RCT were asked for additional permission to audio-record a treatment decision-making encounter with their oncologist. These appointments all included a time in which there was a discussion about changing the current medical treatment (e.g., chemotherapy, hormone therapy, or targeted therapy) to another medical treatment or no treatment. These appointments typically involve substantially more interactive discussion as the multitude of breast cancer treatment options available lends well to patient-centric treatment decision-making. Patients eligible for this sub-study included women aged 18 and older who were diagnosed with and receiving treatment for MBC at the University of Alabama at Birmingham (UAB). Only patients who were accompanied by a caregiver to the recorded appointment were included in this analysis and only one encounter was analyzed per dyad. Demographic and clinical data were extracted from the parent RCT, including age, race, and home address (utilized to calculate the distance traveled to the clinic). Time traveled to the clinic was deemed to be important in order to contextualize potential logistical burdens for patients and caregivers. This study complies with the Declaration of Helsinki and was approved by the UAB Institutional Review Board (IRB-300002283).

### Qualitative data: caregiver actions and quality of participation

Treatment decision-making encounter recordings were transcribed by an independent transcription service and verified by the study team. To facilitate the exploration of the triadic relationship between the oncologist, the patient, and the caregiver, Wolff and Roter [23] posited the family involvement in the interpersonal health processes model that emphasizes relational rapport, information exchange, decision making, and goal setting. We utilized this framework to conduct a focused analysis of the triadic communication that occurs during treatment decision-making visits for women with metastatic breast cancer. A PhD medical anthropologist (NH) and medical resident (GB) independently performed a conventional content analysis, coding each verbal contribution of the caregiver according to the specific action they were performing using NVivo software. After the first round of open coding, coders worked in conjunction with the Principal Investigator (GR) to identify major themes and distinct actions to add to the formal codebook of caregiver participation quality.

We then utilized the formal codebook to conduct a summative thematic content analysis in order to operationalize and quantitatively measure the quality of caregiver participation [59]. For each of the 12 distinct actions (provision of general support; management of medication/treatment; aid in treatment history recall; decision-making; insurance of money; pharmacy; appointment or treatment scheduling; travel concerns; questioning about cancer in general; and questioning about patient-specific cancer biology), caregivers were given a 0 or a 1, depending on whether they had performed the action during the recorded appointment. We then operationalized caregiver participation quality as the total number of actions the caregiver performed during the decision-making encounter (0–12). Each coder summed actions independently for each participant, facilitating the use of inter-coder correlations to establish inter-coder reliability. The correlation between the two coders’ computation of the quality measure was quite strong (*r* (58) = 0.989, *p* < 0.001), indicating robust interrater reliability. Finally, the sample was dichotomized at the mean to identify those patients with “low caregiver participation quality” and “high caregiver participation quality.” This enabled a direct comparison between the effects of the quality and quantity of caregiver participation on patient activation.

### Quantitative data: quantity of caregiver participation

The quantity of caregiver participation was operationalized through timecoding of the recorded appointments. The recording began when the oncologist entered the room and stopped the recording once their encounter with the patient was complete. In each appointment, the treatment decision point was defined as the time when the patient and provider reached a consensus on the next step in the individual’s treatment plan. In some cases, an oncology fellow, pharmacist, or other healthcare professional was involved in the conversation, but these sections were only included in the time analysis if they occurred *before* the treatment decision. For example, oncology fellows often met with patients prior to the oncologist to ascertain their medical history and current status. The fellows would then relay this information to the oncologist, thereby shortening the amount of time that the oncologist needed to spend on the appointment. Conversely, the timing of pharmacists in the encounters was variable. Some pharmacists discussed treatment options prior to the treatment decision point, while others entered the conversation once a definitive plan had been established. In these latter cases, the patient’s discussion with the pharmacist was excluded. Measured time-related variables included total appointment time, as well as total speaking time for the patient, caregiver, oncologist, and other health professionals. Proportion of speaking time was then calculated by dividing each individual’s speaking time by the total appointment time. Again, caregiver speaking time was dichotomized at the mean to identify patients with “low caregiver participation quantity” and “high caregiver participation quantity.”

### Integration: effect of quantity and quality of caregiver participation on patient activation

Descriptive statistics, including frequencies, means, standard deviations (SDs), and ranges for caregiver quality and quantity of participation were calculated. The dichotomized caregiver quality and quantity of participation measures were cross-tabulated to identify proportions of both quantity and quality of caregiver participation. This resulted in four subgroups: low quality/low quantity, low quality/high quantity, high quality/low quantity, and high quality/high quantity.

The patient’s level of engagement in their healthcare was assessed using the Patient Activation Measure (PAM), a 13-item questionnaire that assesses patients’ knowledge, skill, and confidence in managing their own health and healthcare. PAM is scored 1–100, with higher scores representing higher patient activation [57]. PAM was measured 1-month post-treatment decision either electronically through a REDCap Survey or in person on paper, which was then transcribed to the REDCap database. Caregiver participation quality and quantity were then compared to patient activation levels independently through *t*-tests and in combination through an error bar chart.

## Results

### Sample characteristics

Fifty-three patients were accompanied by a caregiver to their recorded decision-making appointment. Demographic information for these patients is available in Table 1. Patients with caregivers were a mean of 57 years old (SD 11) and most often White (70%). Almost all patients were diagnosed with recurrent MBC (90%), and 47% traveled over an hour to receive care at UAB. Demographic information was not collected for caregivers, but 35% of caregivers were verbally identified as the patient’s spouse or partner; 27% were identified as friends, sisters, daughters, or other family members; and 38% of caregivers were not identified relationally during appointments.

**Table 1:** Caregiver identity and demographics of patients (N = 53)

	Total sample *N* (%)
Caregiver identity: partner/spouse	21 (40%)
Other identified*	16 (30%)
Unidentified	16 (30%)
Patient age: under 50	11 (21%)
51–65	31 (58%)
66 and older	11 (21%)
Patient race: white	38 (72%)
Black	15 (28%)
Minutes traveled to appt: less than 30	12 (23%)
30– 1 h	13 (25%)
Over 1 h	23 (43%)
Missing	5 (9%)

^*^Includes friends, siblings, and children of patients

### Caregiver distinct actions

The majority of caregivers (85%) participated in the treatment decision-making conversation at least once. Twelve distinct actions (Table 2) were performed by caregivers in the sample within the larger themes of caretaking (79%), treatment decision-making (70%), managing of logistical concerns (64%), facilitation of cancer understanding (55%), and participation in symptom discussion (55%).

**Table 2:** Major themes and minor actions performed by caregivers during clinical decision-making encounters

Major themes	*N* (%)	Minor actions	*N* (%)	Exemplary quote/interaction
Caretaking	42 (79%)	Provision of general support	34 (64%)	**Caregiver**: “It’s great news about your scans. That’s wonderful.”
		Management of medication/treatment	32 (60%)	**Caregiver**: “She had mentioned that possibly she would have to go off of her arthritis medicine. Is that still the case or what’s going on with that?” **Oncologist**: So, you [patient] probably are going to come off your Methotrexate
		Aid in treatment history recall	23 (43%)	**Caregiver**: “After her fifth round is when she fell and broke her hip and had to have a hip replacement.”
Decision-making	37 (70%)	Participation in decision-making	37 (70%)	**Caregiver**: “What is the success rate of these, that you're talking about?” **Oncologist**: “So, i mean…” **Caregiver**: “Because she’s had so much treatment and continues to mutate… After she had a treatment one time, I don’t know, we had a discussion on, hell, the treatment’s worse than the disease.”
Management of logistical concerns	34 (64%)	Management of insurance or money	9 (17%)	**Caregiver**: “Now, when you do the scans, like three months apart, does insurance cover it?” **Oncologist**: “Yeah. They cover all that.”
		Management of pharmacy	10 (19%)	**Caregiver**: “Now the medications, will I be picking them up here or would there be a pharmacist?”
		Management of appointment or treatment scheduling	25 (47%)	**Caregiver**: “So we come next week, get labs, and then wait to see what they are before we get the shot next week, right?”
		Management of travel concerns	21 (40%)	**Caregiver**: “So it’ll be, come up here, spend the night, come in that morning to have it done, then go home?”
Facilitation of cancer understanding	29 (55%)	Questioning about cancer in general	7 (13%)	**Oncologist**: “My gut instinct is that you may actually have a germline mutation here. Something that sort of predisposed you a little bit to get cancer. It’s not a guarantee that somebody’s going to get cancer, it just means it’s a little more likely if they have something that they’ve had since birth.” **Caregiver**: “Kind of like a cancer gene?” **Oncologist**: “Exactly, a cancer gene. I don’t know that for sure, but there are several things on here that are pretty common, and they’re common to see sort of spontaneously, but they’re also pretty common to see people who sort of have it from birth. I don’t know, I’ll send your official genetics referral so that they can look at that.”
		Questioning about patient’s specific cancer biology	27 (51%)	**Caregiver**: “You just talked about the cancer there, it’s in the bone area, other parts beside the breast. Do you know about what period of time, we’re talking about, that it took for that to happen?”
Participation in symptom discussion	29 (55%)	Caregiver-initiated questioning or emphasis on symptom severity	23 (43%)	**Caregiver**: “I thought of one. You talked about her moving around a little bit. One thing that keeps her from moving around is her pain in her hip and in her ribs.”
		Caregiver back-up of patient symptom description	20 (38%)	**Oncologist**: “Yeah. No fevers or chills? Okay. Any chest pain with that? You feeling short of breath at all?” **Patient**: “If I walk a long ways.” **Caregiver**: “Yeah, when she’s walking.”

### Caretaking

Nearly 80% of caregivers in the study provided patient-related background information or performed care-taking actions during the appointment. These statements took three forms: (1) provision of general support, (2) management of medication/treatment, and (3) aid in treatment history recall. Instances of general support were performed by 64% of the caregivers and included statements demonstrating that the caregiver supports the patient as an individual person, both inside and outside of their treatment management. For example, caregivers described their relationship to the patient (“I’m X’s husband”), provided encouragement, or discussed shared responsibilities at home (e.g., cleaning, cooking meals). Caregivers also contributed considerably to discussions of medical caretaking, with over 60% asking questions related to current or future medications or treatment and over 40% aiding in the recall of the patient’s treatment history. Management questions revolved around the frequency, amount, and timeline of treatments. Caregivers also aided in the recall of treatment history by providing names and timelines of previous medications, treatment plans, or providers.

### Decision-making

The second major component of caregiver advocacy was their contributions to the treatment decision-making process (70%; 4). Often, these inputs were questions about potential treatment options, including how they work, the likelihood of success, their side effects, and the provider’s opinion about the best choice for the patient. Questions regarding accessibility or logistics of new medications were also included here if they were regarding the comparison of one potential treatment to another. Finally, explicit encouragement towards specific treatments and discussions of advanced directives/power of attorney were also included under the decision-making action.

### Managing of logistical concerns

Sixty-four percent of caregivers contributed to discussions regarding logistics making it the third major component. These discussions included the specific actions of (5) appointment/treatment scheduling (47%), (6) concerns (40%), (7) pharmacy (19%), and (8) insurance or finances (17%). Caregivers often helped patients recall when appointments with other members of their healthcare team were scheduled and offered input on future times that would potentially be good for themselves and/or the patient. Travel concerns then encapsulated any mention of the time/distance necessary for the patient to travel in order to receive care. It is important to mention that these statements often served two purposes, either to advocate for a more efficient use of the patient’s time by better organizing their schedule or by expressing their (the caregiver’s) willingness to travel greater distances or more often in order to better support the patient in their care. In either case, the caregivers were clearly aware of the travel burden and were working to alleviate its potential stress on the patient. Some caregivers also discussed pharmacy options, including questioning where specific medications would be filled or stating that they are involved in obtaining the medication for the patient. And, finally, caregivers would ask financial- or insurance-related questions that would often prompt the involvement of another care team member than the oncologist.

### Facilitation of cancer understanding

The fourth most common action was related to cancer understanding, which was discussed by 58% of caregivers. These discussions were further subdivided into (9) questions/statements regarding the understanding of how cancer works in general (13%) versus (10) seeking clarification or more information about the patient’s cancer specifically (51%). In each of these incidences, the purpose of the caregiver’s participation was to improve either their own or the patient’s understanding of their diagnosis. These typically occurred at the beginning of the appointment when the oncologist was reviewing new and old scans and discussing why a treatment change was potentially necessary.

### Participation in symptom discussion

The final major action involved the discussion of patient symptoms and side effects (55%). These contributions were subdivided into two categories based on whether the discussion was initiated by (11) the patient (38%) or (12) the caregiver (43%). In the former case, the caregiver acted as a backup or support to the patient, confirming that the patient’s presentation of symptoms was accurate. In other cases, however, it was the caregiver that prompted the discussion, or they actively disagreed with the patient’s characterization of their wellbeing. In each of these cases, the caregiver argued that various symptoms/side effects were a *greater* burden to the patient and sought the oncologist’s opinion about what could be done to better manage the experience.

### Caregiver participation quality

Based on the actions identified from the summative content analysis, a range of 0 through 12 potential actions were displayed, where 0 represented no contributions and 12 represented more diverse participation during the decision-making encounter. The mean number of minor actions performed during the decision-making encounter was 5 (SD 3). Of caregivers present, eight (15%) did not perform any actions and only one performed every identified action during the clinical encounter. A slight majority of patients (52%) had caregivers with high-quality participation, while 48% had caregivers with low-quality participation.

### Caregiver participation quantity

The average appointment time for all patients was 29 min (SD 13), with oncologists speaking for an average 76% of the decision-making encounter. Patients were the next most common contributor, speaking an average of 20% of the total appointment time, while caregivers contributed an average of 4% of the conversation. A majority of patients (60%) had caregivers with low-quality participation, while 40% had caregivers with high-quantity participation.

### Effect of quantity and quality of caregiver participation on patient activation

When combining caregiver participation quality and quantity (Table 3), the largest subgroup of patients (42%) had caregivers with both low quality and quantity of participation, 34% had both high quality and quantity of participation, 18% had high quality and low quantity of participation, and 6% had low quality and high quantity of participation.

**Table 3:** Crosstabulation of quality and quantity of caregiver participation

		Quantity of caregiver participation	
		Low	High
Quality of caregiver participation	Low	42% (22 patients)	6% (3 patients)
	High	18% (10 patients)	34% (18 patients)

**Fig. 1:**
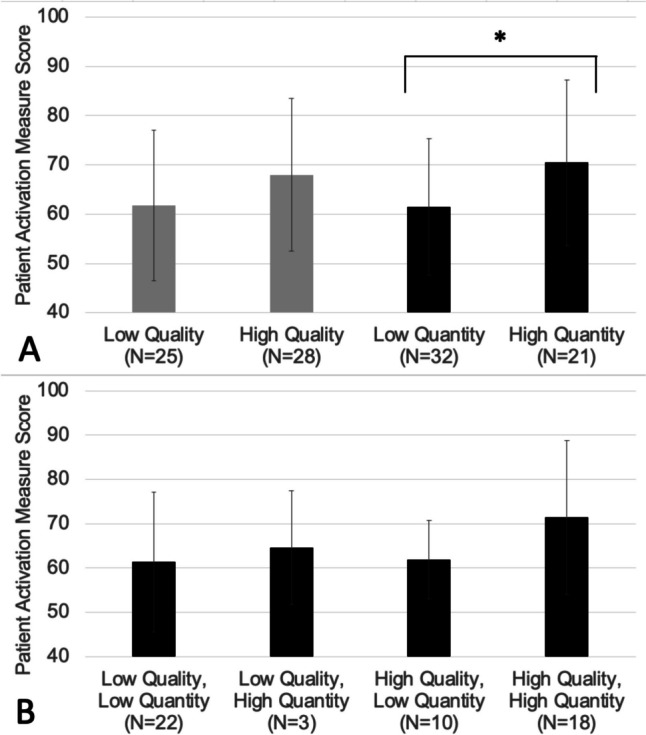
**A** and **B** Means and standard deviations of patient activation by quality and quantity of caregiver participation (*N* = 53), (* = statistically significant difference at the alpha of 0.05 level)

According to validated cut-points, most patients (82%) reported a moderate to high level of activation (PAM score range 42–100, mean 65, SD 16). Patients with both low and high quality of caregiver participation had similar levels of patient activation (low, PAM score 62, SD 15; high, PAM score 68, SD 16; *p* = 0.07). Patients with high quantity of caregiver participation had slightly higher levels of patient activation when compared to those with low quantity of caregiver participation (high, PAM score 71, SD 17; low, PAM score 62, SD 14; *p* = 0.02; Fig. 1A). When examining the combined association of both quality and quantity of caregiver participation on patient activation (Fig. 1B), patients with both low quality and quantity of caregiver participation had the lowest mean PAM score (61, SD 16), while patients with both high quality and quantity of caregiver participation had the highest mean PAM score (71, SD 17). However, this difference was not statistically significant [F(3,49) = 1.625, *p* = 0.196].

## Discussion

This study demonstrated that the quality and quantity of caregiver participation during decision-making visits for women with MBC positively impacted patient activation. We utilized conventional and summative thematic content analysis to operationalize the quality of caregiver participation, defined as the number of minor actions performed by the caregiver during the appointment. These actions were subdivided into five major categories and included: General Support; Management of Treatment or Medication; Treatment History; Decision-Making; Insurance or Money; Pharmacy; Scheduling; Travel Concerns; General Cancer Understanding; Patient Specific Cancer Understanding; Caregiver-Initiated or Emphasis on Symptom Severity; and Caregiver Back-Up of Patient Symptom Description. This enabled us to capture multiple aspects and levels of caregiver support, ranging from their mere presence to their participation in conversation, to the tangible performance of patient advocacy and aid during the clinical encounter.

Among caregivers, the quantity of conversation participation seemed to impact the quality of caregiver participation, in that the more they spoke, the more likely they were to perform diverse actions. However, this association was not perfectly consistent. Some caregivers were quite talkative but primarily discussed clinically irrelevant topics or did not keep the focus on the patient themself. Others rarely spoke during the appointment, but when they did, it was to offer clinically meaningful information. This interaction between quality and quantity highlights the importance of delving into the content of appointment discussions, as the more ways that the caregiver productively participates in the clinical encounter, the more empowered the patient is and the more comfortable and knowledgeable they feel in their treatment.

This finding adds to the emerging literature on the diverse characteristics of caregivers’ involvement and influence on patients [2, 16, 26]. According to Acquati and colleagues, low caregiver involvement affects a patient’s adherence to and persistence with the treatment regime, irrespective of patient activation [26]. The level of caregiver involvement is further extended to influencing patient’s decision-making, adherence to treatment, and practice of healthful behaviors [60–62]. Furthermore, research on identity and relational needs in clinical settings educates this finding on caregiver contributions to appointment discussions and the patient decision-making process. Both Krieger and colleagues and Venetis and colleagues highlight the sensitivity to the caregiver and patient illness identity and relationship needs that influence caregiver involvement in clinical settings [63, 64]. This suggests focusing on interventions that address triadic interaction that includes caregiver participation in clinical settings [16, 64].

### Clinical implication

Caregivers not only provide tangible and useful clinical information for healthcare providers during appointments but also act as support systems and advocates for the patients. Caregivers have a unique perspective on the patient’s health and needs and can provide valuable information to healthcare providers that could increase their quality and satisfaction of care. Greater integration of the caregiver into decision-making conversations can help to ensure that the patient’s preferences and values are considered when developing a treatment plan and can improve communication and collaboration between healthcare providers and the patient’s support system. Physician recognition of the importance of these roles and movement towards greater integration of the caregiver into clinical encounters could therefore facilitate better understanding, agency, and satisfaction with the patient’s treatment experience.

With the significant role caregivers play in patient activation, findings inform the need for the utilization of communication and health literacy resources to shape patient activation via caregiver education [65, 66]. The *eTRIO* intervention protocol by Juraskova and colleagues engages with supporting and education caregivers with communication skills and self-efficacy in supporting patients [67]. Informed, supported, and less psychological distress caregiver instills confidence, and cognizant engagement in caregivers to better interact with patients and clinicians. Another avenue of patient and caregiver support in decision-making is through psychosocial interventions. These interventions are designed for effective communication and relationship maintenance between patients and caregivers regarding respective treatment associated choices, responsibilities, and limitations [47, 68]. This study provides further support for the necessity of the widescale implementation of evidence-based interventions that educate and build communication and shared decision-making skills among patients, caregivers, and providers.

### Limitations

The limitations of this study are similar to other qualitative and mixed methods studies in that the sample was relatively small and constructed through convenience sampling methods. These data were collected in the context of a trial testing an intervention to facilitate shared decision-making between patients and physicians, which may have impacted how the caregiver engaged in the conversation. However, significant findings were lacking in the parent trial and thus not expected to have a substantial influence on caregiver engagement. The sixty recorded appointments were less than half of the initial 126 patients who consented to appointment recording; however, logistical challenges including visit conversion to telehealth during the COVID-19 pandemic and physicians not remembering to record limited the sample. Data were also collected from a single institution during a limited timeframe, which means that the sample may be non-representative of other physician, patient, and caregiver populations. Caregiver mood, energy level, health literacy, and context also were not assessed and may have impacted the quality and quantity of caregivers’ participation. Finally, patient-caregiver relationships outside of the clinical context were not assessed and the strength of this relationship could also impact results.

## Conclusions

Caregivers perform a variety of tasks and actions during oncological decision-making clinical visits that aid both the patient and the provider. Greater participation in terms of quantity and quality by the caregiver was associated with greater patient activism, indicating a need for better integration of the caregiver in the clinical decision-making environment. More research is needed regarding how best to incorporate caregivers while maintaining the patient’s preferences and agency in the clinical encounter.

## Data Availability

The data that support the findings of this study are available from the senior author, GBR, upon reasonable request.
